# Impact of Postoperative Radiotherapy on the Risk of Ischemic Heart Disease and Survival in Patients with Ductal Carcinoma In Situ: A Nationwide Claims-Based Cohort Study

**DOI:** 10.3390/cancers17233805

**Published:** 2025-11-27

**Authors:** Van Hung Nguyen, Seung Yeun Chung, O Kyu Noh

**Affiliations:** 1 Oncology Center, Hanoi Medical University Hospital, Hanoi 100000, Vietnam; 2 Department of Radiation Oncology, Ajou University School of Medicine, 164 Worldcup-ro, Yeongtong-gu, Suwon 16499, Republic of Korea

**Keywords:** ductal carcinoma in situ, radiotherapy, ischemic heart disease, overall survival, real-world data

## Abstract

Background: Postoperative radiation therapy (RT) after lumpectomy reduces the risk of locoregional recurrence in ductal carcinoma in situ (DCIS). However, the potential association between RT and ischemic heart disease (IHD) remains uncertain. This nationwide cohort study evaluated the long-term impact of postoperative RT on IHD risk and overall survival (OS) in women with DCIS using real-world data from the Korean National Health Insurance Service (NHIS). Methods: Women diagnosed with DCIS who underwent breast-conserving surgery between 2003 and 2020 were identified from the NHIS claims database. Patients with invasive breast cancer, age under 20 years, a prior history of IHD, or missing smoking or body mass index (BMI) data were excluded. Multivariable Cox regression was performed to assess the association between postoperative RT, IHD incidence, and OS, adjusting for key cardiovascular risk factors. Results: Among 4633 eligible patients (RT, 2778; no RT, 1855), the median follow-up duration was 86.1 months, and baseline characteristics were well balanced between groups without major differences in cardiovascular risk factors. A total of 126 patients (3.4%) developed IHD, with a 10-year cumulative incidence of 4.7%. Older age, hypertension, and hyperlipidemia were independent risk factors for IHD, whereas postoperative RT was not significantly associated with increased IHD risk (hazard ratio [HR] = 1.07, 95% confidence interval [CI] = 0.77–1.48; *p* = 0.690). The 10-year OS rate was 98.0%, and postoperative RT remained an independent predictor of improved survival (HR = 0.47, 95% CI = 0.28–0.79; *p* = 0.004). Conclusions: Postoperative RT did not increase the long-term risk of IHD but was associated with improved OS in patients with DCIS. These findings provide population-based evidence supporting the cardiac safety and oncologic efficacy of postoperative RT, while recognizing that unmeasured differences in health behavior or medical care utilization could have contributed to the observed survival benefit.

## Simple Summary

Ductal carcinoma in situ is an early form of breast cancer that is usually treated with breast-conserving surgery. Many patients receive radiation therapy afterward to reduce the chance of the disease returning. Some people worry that radiation might increase the risk of heart disease in the long term. In this study, we used nationwide health insurance data from Korea to compare women who received radiation therapy with those who did not. We found that radiation therapy did not increase the long-term risk of ischemic heart disease. Women who received radiation also showed better long-term survival, although this result may be related to differences in health habits or medical follow-up. Overall, our findings suggest that radiation therapy after surgery is generally safe for the heart in this condition. More research using modern radiation techniques and longer follow-up will help guide future treatment decisions.

## 1. Introduction

Ductal carcinoma in situ (DCIS) is a non-invasive breast malignancy characterized by the proliferation of abnormal ductal epithelial cells confined within the mammary ductal system. The incidence of DCIS has increased with the widespread adoption of mammographic screening, now accounting for approximately 15% of newly diagnosed breast malignancies [1,2]. Although DCIS is generally associated with an excellent prognosis, a subset of cases may progress to invasive breast cancer, highlighting the importance of appropriate local management and long-term surveillance.

The standard management includes breast-conserving surgery (BCS), and postoperative radiotherapy (RT) has been shown to reduce the risk of ipsilateral breast recurrence by approximately 50% compared to surgery alone [3,4,5]. However, RT inevitably exposes part of the heart to radiation. Such exposure may increase the long-term risk of ischemic heart disease (IHD) [6]. In patients with invasive breast cancer, multiple studies have demonstrated a dose-dependent relationship between RT exposure and IHD risk [7,8,9]. These findings have reinforced the importance of optimizing RT techniques to minimize cardiac exposure while maintaining effective local control. Advances in RT delivery have significantly reduced cardiac radiation exposure, suggesting that modern RT techniques may not substantially increase IHD incidence [10]. However, these findings are primarily based on studies involving patients with invasive breast cancer, limiting their direct applicability to DCIS. Because DCIS patients generally receive lower radiation doses and do not undergo cardiotoxic chemotherapy, the risk of RT-induced IHD is presumed to be lower.

Direct evidence of RT-associated cardiac toxicity in DCIS is limited, particularly in Asian populations. Population-based studies from Sweden and the Netherlands found no significant increase in ischemic heart disease or cardiovascular mortality after RT in DCIS patients [11,12]. A small single-institution study from Korea also reported no excess cardiac morbidity, though limited by sample size and follow-up [13]. These findings suggest minimal cardiac risk after RT in DCIS, yet data in Asian populations remain scarce, warranting further investigation using nationwide cohorts.

Despite the clinical and treatment-related differences between DCIS and invasive breast cancer, the true impact of postoperative RT on IHD risk in DCIS remains unclear, because real-world clinical practice differs from controlled trial settings, and the distribution of cardiovascular risk factors may vary. Furthermore, because DCIS has an excellent prognosis, both patients and caregivers often question the necessity of RT without robust evidence of its long-term risks and benefits. Identifying which subgroups of DCIS patients benefit from RT remains a critical issue.

Regarding overall survival (OS), randomized clinical trials have shown no significant OS benefit of postoperative RT in DCIS patients [3,4,5]. However, it remains uncertain whether these findings translate to broader patient populations in real-world settings. Moreover, as improvements in early detection and longevity have led to an expanding population of long-term DCIS survivors, the potential late toxicity of RT—particularly cardiac complications—has become an increasingly relevant clinical concern. In this context, modern treatment decisions must balance the established oncologic benefits of RT against possible long-term cardiovascular risks.

The National Health Insurance Service (NHIS) database in South Korea is a comprehensive public healthcare resource that covers almost the entire population through insurance claims data [14]. This database enables the nationwide observation of real-world treatment patterns and clinical outcomes across diverse healthcare settings. Leveraging the NHIS database, this study aims to assess the real-world impact of postoperative RT on IHD risk and OS in DCIS patients, comparing outcomes between those who received RT and those who did not. This nationwide population-based study provides robust, real-world evidence to clarify the long-term risks and benefits of postoperative RT in patients with DCIS.

## 2. Materials and Methods

### 2.1. Study Population

This retrospective cohort study utilized data from the Korean National Health Insurance Service (NHIS) claims database between January 2003 and December 2020. The NHIS is a population-based nationwide healthcare system covering nearly the entire Korean population and provides comprehensive information on diagnoses, procedures, prescriptions, and demographic characteristics. The database includes longitudinal records that enable continuous tracking of healthcare utilization, diagnoses, and outcomes for individual over time. Each patient was followed from the index date until the occurrence of IHD, death, or the end of the study period (31 December 2020), whichever came first. The median duration of follow-up and its interquartile range were calculated to assess the temporal completeness of the dataset.

Using the International Classification of Diseases, Tenth Revision (ICD-10) code D05.x, we identified women diagnosed with DCIS who underwent BCS. The date of BCS was defined as the index date. To ensure accurate case identification, patients with a history of invasive breast cancer (C50.x) or ischemic heart disease (I20–I25) before DCIS diagnosis were excluded. Additional exclusion criteria were age under 20 years, receipt of preoperative or postoperative chemotherapy, missing data on smoking history or body mass index (BMI), and incomplete follow-up. The proportion of missing values for BMI and smoking status in the NHIS screening database is extremely low because these variables are collected for nearly the entire adult population. Patients with missing values represented only a very small fraction and were excluded to ensure accurate adjustment for cardiovascular risk.

**Figure 1:**
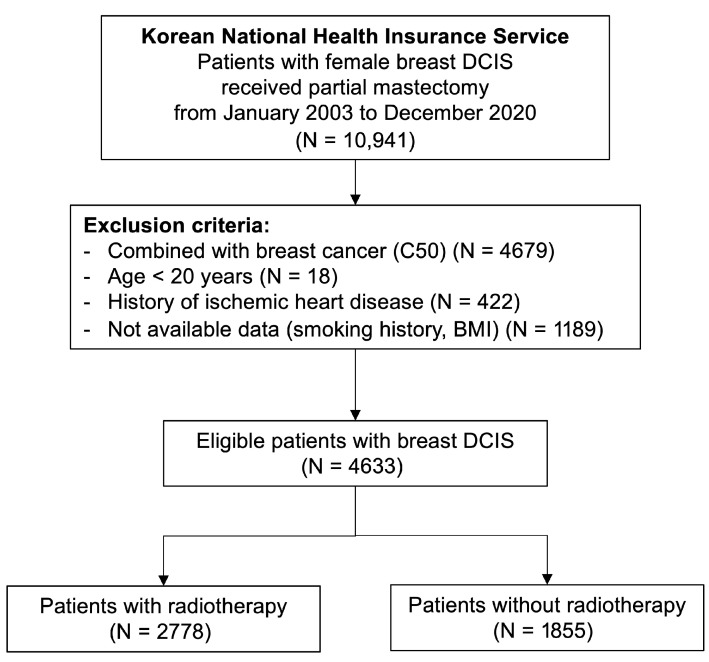
Study Selection Flowchart.

After applying these criteria, a total of 4633 women were eligible for analysis, including 2778 who received postoperative RT and 1855 who did not. Baseline demographic and clinical characteristics, including age and comorbidities (hypertension, diabetes mellitus, and dyslipidemia), were obtained. Comorbidities were identified based on at least two outpatient claims or one inpatient claim with the corresponding ICD-10 codes within one year before the index date. Smoking status was categorized as never, former, or current smoker, and BMI was classified according to the WHO Asian criteria, with obesity defined as BMI ≥ 25 kg/m^2^. Information on smoking status and BMI was derived from the NHIS health-screening database when available. The study selection process is summarized in Figure 1.

### 2.2. Statistical Analysis

Continuous variables were expressed as mean ± standard deviation (SD) or median with interquartile range (IQR), while categorical variables were summarized as counts and percentages. Differences between groups were evaluated using Student’s *t*-test or the Mann–Whitney U test for continuous variables and the Chi-square test or Fisher’s exact test for categorical variables. The cumulative incidence of IHD was estimated using the Fine–Gray competing-risk model, treating death from any cause as a competing event, and group comparisons were performed using Gray’s test. OS was evaluated using the Kaplan–Meier method and compared using the log-rank test.

Adjusted hazard ratios (HRs) and 95% confidence intervals (CIs) for OS and IHD were calculated using Cox proportional hazards regression and Fine–Gray subdistribution hazard models, respectively. Covariates included in the multivariable models were age, BMI, smoking status, hypertension, diabetes mellitus, and dyslipidemia. Age and BMI were treated as continuous variables, and smoking status and comorbidities were coded as binary categorical variables. The same coding scheme was applied consistently in both univariable and multivariable Cox models. Covariate selection was based on established clinical relevance and previous literature on cardiovascular risk after breast radiotherapy. Proportional hazards assumptions were evaluated using Schoenfeld residuals, and no significant violation was detected. The Fine–Gray model was also cross-validated with cause-specific hazards regression to ensure the robustness of the direction and magnitude of associations. Sensitivity analyses were conducted to assess the robustness of the findings with respect to follow-up duration and missing health-screening data.

All analyses were performed using R software, version 4.2.0 (R Foundation for Statistical Computing, Vienna, Austria), employing the cmprsk and survival packages. A two-sided *p* value < 0.05 was considered statistically significant.

### 2.3. Ethics Statement

This study was approved by the Institutional Review Board of Ajou University Hospital (approval number: AJUIRB-EXP-2019-359) and the NHIS Data Provision Review Committee. Because the data were fully anonymized, the requirement for written informed consent was waived. All data analyses were performed within the secure remote-access environment provided by the NHIS to ensure compliance with national data protection and privacy regulations.

## 3. Results

### 3.1. Patient Characteristics

A total of 10,941 women diagnosed with DCIS who underwent partial mastectomy between January 2003 and December 2020 were identified from the Korean NHIS database. According to predefined exclusion criteria, 4633 patients comprised the final analytic cohort (Figure 1). Among them, 2778 (60.0%) received postoperative RT, while 1855 (40.0%) did not. The median follow-up duration for the entire cohort was 86.1 months (IQR, 57.4–119.4 months). Baseline demographic and clinical characteristics were well balanced between the RT and non-RT groups, with no significant differences in age, comorbidities, or cardiovascular risk factors (Table 1).

### 3.2. Overall Survival

The 10-year OS rate for the entire cohort was 98.0%. In the univariable analysis, postoperative RT was significantly associated with improved OS (HR = 0.47, 95% CI: 0.28–0.79, *p* = 0.004), whereas older age and higher body mass index were correlated with poorer survival (Table 2).

After adjustment for potential confounders, RT remained an independent predictor of improved survival (HR = 0.47, 95% CI: 0.28–0.79, *p* = 0.004). Conversely, older age was independently associated with increased mortality risk (HR = 1.07 per year, 95% CI: 1.04–1.10, *p* < 0.001). Other comorbidities, including hypertension, diabetes, and dyslipidemia, were not significantly related to OS.

**Figure 2:**
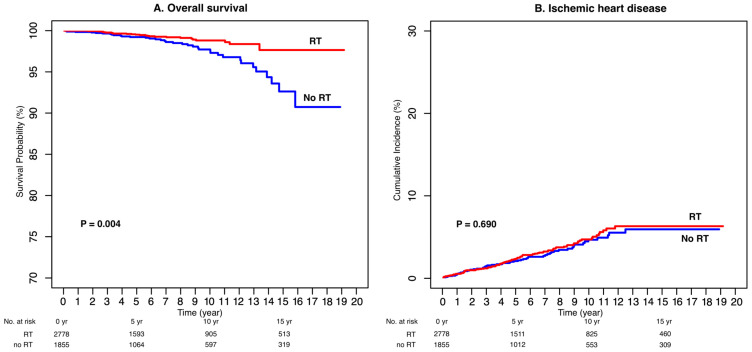
Overall Survival (**A**) and Cumulative Incidence of Ischemic Heart Disease (**B**).

These findings indicate that, despite the excellent overall prognosis of DCIS, adjuvant RT provides a measurable long-term survival advantage, even after accounting for age and metabolic risk factors (Figure 2A).

### 3.3. Ischemic Heart Disease

Throughout the observation period, 126 patients (3.4%) developed IHD, corresponding to a 10-year cumulative incidence of 4.7%. No significant difference in IHD incidence was observed between the RT and non-RT groups (*p* = 0.690; Figure 2B).

In the multivariable Fine–Gray competing-risk model, older age (HR = 1.04 per year, 95% CI: 1.02–1.05, *p* < 0.001), hypertension (HR = 1.58, 95% CI: 1.08–2.30, *p* = 0.020), and hyperlipidemia (HR = 1.71, 95% CI: 1.08–2.70, *p* = 0.022) were identified as independent risk factors for IHD (Table 3). However, RT exposure was not significantly associated with increased IHD risk (HR = 1.07, 95% CI: 0.77–1.48, *p* = 0.690).

Taken together, these findings suggest that the cardiac safety of postoperative RT in DCIS is acceptable, and that IHD risk is mainly driven by baseline cardiovascular comorbidities rather than radiation exposure.

## 4. Discussion

This nationwide population-based cohort study evaluated the long-term effects of postoperative RT on IHD and OS in patients with DCIS. Using real-world data from the Korean NHIS, RT was not associated with an increased risk of IHD, whereas OS was significantly improved among patients who received RT.

The absence of excess IHD risk among RT-treated patients may be explained by the evolution of standard radiotherapy techniques, which have effectively reduced unnecessary cardiac exposure [10,15]. Intensity-modulated radiotherapy (IMRT) and deep inspiration breath-hold (DIBH) are well-established methods for minimizing cardiac dose [16,17]. Moreover, patients with DCIS, unlike those with invasive breast cancer, do not receive cardiotoxic chemotherapy and are typically treated with lower total radiation doses [18]. Because DCIS is often diagnosed at an earlier age, these patients generally have lower baseline cardiovascular risk, which may further attenuate the incremental impact of RT on IHD. Collectively, these factors likely contribute to the very low cumulative incidence of cardiac disease observed in this population. In addition, cardiac-sparing strategies such as prone positioning, partial-breast irradiation, and image-guided adaptive RT are increasingly implemented in clinical practice, suggesting that the long-term cardiovascular risk of RT will continue to decrease.

Meanwhile, the transition from three-dimensional conformal radiotherapy (3D-CRT) to IMRT and volumetric modulated arc therapy (VMAT) has altered the pattern of cardiac dose distribution [19]. While tangential 3D-CRT fields generally avoid direct heart exposure, focal regions of high-dose irradiation can occasionally occur. In contrast, IMRT and VMAT markedly reduce these high-dose areas but tend to increase the volume of low-dose exposure (V5–V10 Gy) [20]. Although the clinical significance of this “low-dose bath” remains uncertain, several reports suggest that it may contribute to late-onset IHD, particularly in patients with preexisting cardiovascular comorbidities such as hypertension or dyslipidemia [9,21]. Importantly, most patients in our study were treated before the widespread adoption of IMRT and VMAT, when 3D-CRT represented the standard technique. Thus, our findings can be interpreted as a conservative estimate of cardiac risk under conventional RT. In more recent cohorts treated with advanced techniques, the mean heart dose is expected to be even lower. Future studies incorporating detailed dosimetric data and extended follow-up are warranted to quantitatively assess these evolving trends.

Previous randomized controlled trials (RCTs) have reported no significant improvement in OS following RT for DCIS [3,4,5], likely reflecting the inherently favorable prognosis of DCIS and the effectiveness of salvage treatments for local recurrence. In contrast, our study demonstrated a significant survival advantage among RT-treated patients. This discrepancy may result from differences in study design and patient behavior. Whereas RCTs minimize selection bias through randomization, observational studies cannot eliminate it. Patients who receive RT tend to be more health-conscious and may participate more actively in regular surveillance and management of comorbidities. This phenomenon, known as the surveillance effect, could lead to earlier detection of non-malignant conditions or secondary cancers, thereby contributing to improved survival [22]. Furthermore, patients who complete RT may represent a group with higher health literacy and greater adherence to preventive healthcare. Although such socioeconomic and behavioral factors cannot be fully captured in claims-based data, they may have partially influenced the observed survival difference. Therefore, the survival advantage in the RT group should be interpreted as a combined effect of true treatment efficacy and enhanced medical surveillance. In this context, the observed OS benefit in our study is unlikely to represent a direct therapeutic effect of RT alone, but rather reflects combined influences of patient health behavior, adherence to follow-up, and differential medical surveillance between groups.

Further analyses using propensity score matching (PSM) or other advanced statistical methods are warranted to minimize selection bias [23]. Recent prospective trials have also suggested that an active monitoring (AM) approach in carefully selected low-risk DCIS patients does not significantly increase the risk of invasive recurrence compared with immediate surgery or RT [24]. These emerging data underscore the importance of a personalized approach to DCIS management and highlight the need for tailored treatment strategies that integrate selective therapy and long-term follow-up. In this context, the improved survival observed in our RT group may reflect not only the biological benefits of RT but also the impact of continued follow-up and preventive medical interventions, providing meaningful guidance for future clinical decision-making.

From a clinical perspective, this study supports the role of adjuvant RT as a safe and effective component of DCIS management. The standard 3D-CRT used during the study period provided durable oncologic control without increasing the risk of cardiovascular complications. In the era of growing numbers of long-term DCIS survivors, these findings may alleviate concerns among patients and clinicians regarding cardiac safety and offer real-world evidence to guide treatment decisions.

This study has several limitations inherent to retrospective analyses of real-world claims data. First, individual dosimetric data were unavailable, and although laterality information exists in the claims database, the proportion of patients with reliably coded left- or right-sided disease was very low. Therefore, a separate analysis by laterality was not feasible and may have limited our ability to fully assess laterality-specific cardiac exposure. Second, information on local recurrence was unavailable, preventing direct assessment of RT’s primary purpose—local control. Third, information on endocrine therapy (tamoxifen or aromatase inhibitors) was not available in the claims dataset. Because endocrine therapy may influence both cardiovascular risk and OS, residual confounding related to endocrine therapy could not be fully addressed. Fourth, although the median follow-up exceeded seven years, it may still be insufficient to fully evaluate very late cardiac toxicity that can occur 10–20 years after RT. Lastly, patients receiving RT may visit hospitals more frequently and undergo regular check-ups, introducing potential observation or surveillance bias. Nevertheless, this study has notable strengths, including the use of a large, population-based dataset that reflects real-world clinical practice.

## 5. Conclusions

This nationwide cohort study demonstrated that postoperative RT was not associated with an increased risk of IHD but was related to improved OS. These findings reinforce the long-term cardiac safety of RT and highlight the need to consider the potential influence of medical surveillance when interpreting survival outcomes. Future studies should include patients treated with more advanced RT techniques, longer follow-up and more comprehensive statistical modeling, to more fully evaluate both oncologic efficacy and cardiovascular safety. Additional evidence from Asian populations will provide valuable real-world data to support clinical decision-making.

## Figures and Tables

**Table 1:** Patients’ characteristics.

Variables	No. of Patients (%)		*p*-Value
	No RT Group (*n* = 1855)	RT Group (*n* = 2788)	
Age (year)			0.082
Mean ± SD	50.4 ± 9.8	50.9 ± 9.1	
Body mass index (kg/m^2^)			0.544
Mean ± SD	23.0 ± 3.3	23.1 ± 3.2	
Smoking history			1.000
Non-smoker	1803 (97.2)	2700 (97.2)	
Current or ex-smoker	52 (2.8)	78 (2.8)	
Diabetes mellitus			0.229
No	1721 (93.1)	2612 (94.0)	
Yes	128 (6.9)	166 (6.0)	
Hypertension			0.219
No	1595 (86.0)	2351 (84.6)	
Yes	260 (14.0)	427 (15.4)	
Hyperlipidemia			0.591
No	1713 (92.3)	2552 (91.9)	
Yes	142 (7.7)	226 (8.1)	

RT = Radiation therapy. Note: Patients with missing baseline variables were excluded during cohort construction; therefore, Table 1 contains complete-case data with no missing values.

**Table 2:** Univariable and multivariable analysis for overall survival.

Variables	Univariable			Multivariable		
	HR	95% CI	*p*-Value	HR	95% CI	*p*-Value
Age	1.08	1.05–1.10	<0.001	1.07	1.04–1.10	<0.001
Radiation therapy	0.47	0.28–0.79	0.004	0.47	0.28–0.79	0.004
Body mass index	1.09	1.02–1.17	0.016	1.02	0.95–1.11	0.557
Current or ex-smoking	0.94	0.13–6.83	0.955	1.25	0.17–9.09	0.825
Diabetes mellitus	2.67	1.27–5.63	0.010	1.35	0.62–2.95	0.457
Hypertension	2.23	1.27–3.91	0.004	1.14	0.61–2.12	0.677
Hyperlipidemia	0.49	0.12–2.00	0.320	0.34	0.08–1.42	0.140

HR = hazard ratio, CI = confidence interval.

**Table 3:** Univariable and multivariable analyses for cumulative incidence of ischemic heart disease.

Variables	Univariable			Multivariable		
	HR	95% CI	*p*-Value	HR	95% CI	*p*-Value
Age	1.05	1.04–1.07	<0.001	1.04	1.02–1.05	<0.001
Radiation therapy	1.09	0.79–1.50	0.612	1.07	0.77–1.48	0.690
Body mass index	1.09	1.04–1.13	<0.001	1.04	0.99–1.09	0.100
Current or ex-smoking	0.87	0.28–2.72	0.808	0.93	0.30–2.92	0.902
Diabetes mellitus	1.96	1.18–3.24	0.009	1.22	0.72–2.06	0.460
Hypertension	2.39	1.70–3.37	<0.001	1.57	1.08–2.30	0.020
Hyperlipidemia	2.15	1.37–3.37	<0.001	1.71	1.08–2.70	0.022

HR = hazard ratio, CI = confidence interval.

## Data Availability

The data that support the findings of this study are available from the Korean National Health Insurance Service (NHIS). Restrictions apply to the availability of these data, which were used under license for this study. Data are not publicly available but may be requested from the NHIS (https://nhiss.nhis.or.kr (accessed on 30 March 2023)) with appropriate institutional approval.
